# Nano-Topographical Control of Ti-Nb-Zr Alloy Surfaces for Enhanced Osteoblastic Response

**DOI:** 10.3390/nano11061507

**Published:** 2021-06-07

**Authors:** Min-Kyu Lee, Hyun Lee, Hyoun-Ee Kim, Eun-Jung Lee, Tae-Sik Jang, Hyun-Do Jung

**Affiliations:** 1 Department of Materials Science and Engineering, Seoul National University, Seoul 08826, Korea; elzkdnem@snu.ac.kr (M.-K.L.); kimhe@snu.ac.kr (H.-E.K.); 2 Department of Materials Science and Engineering and Querrey-Simpson Institute for Bioelectronics, Northwestern University, Evanston, IL 60208, USA; 3 Department of Biomedical-Chemical Engineering, Catholic University of Korea, Bucheon 14662, Korea; leeh0520@catholic.ac.kr; 4 Department of Biotechnology, The Catholic University of Korea, Bucheon 14662, Korea; 5 Department of Nano-Biomedical Science & BK21 PLUS NBM Global Research Center for Regenerative Medicine, Dankook University, Cheonan 31116, Korea; ejlee79@dankook.ac.kr; 6 Department of Materials Science and Engineering, Chosun University, Gwangju 61452, Korea

**Keywords:** Ti-based alloy, ion etching, nano-topography, biocompatibility, biomedical implants

## Abstract

Nano-scale surface roughening of metallic bio-implants plays an important role in the clinical success of hard tissue reconstruction and replacement. In this study, the nano-topographical features of titanium-niobium-zirconium (TNZ) alloy surfaces were controlled by using the target-ion induced plasma sputtering (TIPS) technique to improve the in vitro osteoblastic response. The TIPS technique is a novel strategy for etching the surface of metallic bio-implants using bombardment of target metal cations, which were accelerated by an extremely high negative bias voltage applied to the substrates. The nano-topography of the TNZ surfaces was successfully controlled by modulating experimental variables (such as the ion etching energy and the type of substrate or target materials) of TIPS. As a result, various nanopatterns (size: 10–210 nm) were fabricated on the surface of the TNZ alloys. Compared with the control group, experimental groups with nanopattern widths of ≥130 nm (130 and 210 nm groups) exhibited superior cell adhesion, proliferation, and differentiation. Our findings demonstrate that TIPS is a promising technology that can impart excellent biological functions to the surface of metallic bio-implants.

## 1. Introduction

Ti-based alloys including Ti-6Al-4V are well-established bio-metals that exhibit excellent mechanical strength and biocompatibility required for various applications such as dental implants and bone plates/pins [1,2,3,4]. However, alloying elements of Ti-6Al-4V can induce a cytotoxic response to bone tissue, leading to the failure of medical implants [5]. Titanium-niobium-zirconium (TNZ) alloys are a recently suggested alloy system with a low Young’s modulus that is comparable to that of the cortical bone. Moreover, these alloys exhibit excellent biocompatibility due to the biocompatible alloying elements, niobium (Nb) and zirconium (Zr) [6,7]. Despite these attributes, the rate of osseointegration at the early stage of implantation is still too low to achieve a robust fixation of TNZ implants. The clinical success of bio-implants for hard tissue replacement is determined by the rapid and robust fixation with surrounding host tissue. Accelerated osseointegration between the surface of bio-implants and the surrounding tissue can be achieved through surface treatments, such as chemical functionalization or physical surface modifications, of the implants [8,9,10].

Surface roughening of metals promotes favorable osteoblastic response due to the physical interaction between osteoblasts and micro- or nano-roughened metal surfaces [11,12]. The nano-topography with a size similar to that of the extracellular matrix (ECM) induces rapid reconstruction of impaired tissue by regulating protein interaction and cell behavior. Furthermore, nano-scale roughening of Ti-based alloy surfaces yielded accelerated cell adhesion, proliferation, and differentiation in in vitro cell tests as well as improved bone-to-implant contact values in in vivo animal tests [13]. Several approaches, such as de-alloying, anodizing, and target-ion induced plasma sputtering (TIPS) [14,15,16], have been used to generate nano-topography on metal surfaces. Both de-alloyed Ti-based alloy (Ti-6Al-7Nb) and anodized Ti surfaces enhanced cytocompatibility due to the formation of nanostructures. In spite of these achievements, the complicated and time-consuming process of de-alloying and the poor coating stability between anodic oxidation layer and Ti of anodization process remain as serious limitations of these approaches.

The TIPS technique is a simple yet powerful method of imparting biological functions to metallic bio-implants. This method employs a modified DC magnetron sputtering system that triggers target cation bombardment toward substrates by applying an extremely high negative bias voltage to the substrates. After the TIPS process, the flat substrate became nanostructured via metal ion etching and this structure exhibited excellent biological characteristics including excellent hemocompatibility and osseointegration [17,18]. However, the dependence of nano-topography and cellular behavior on the type of ion etching source remains unclear.

In this study, we present the optimistic potentials of TIPS-treated TNZ surfaces for dental and orthopedic applications. We use niobium (Nb) and tantalum (Ta) metals as target materials in the TIPS system, owing to their excellent biocompatibility and high ion etching efficiency [19,20]. The nano-topography of the TNZ surfaces was optimized by adjusting the experimental parameters (i.e., target metal ion etching energy and the type of target materials) of the TIPS technique. In vitro osteoblastic response of the nanostructured TNZ surfaces was confirmed through a cell attachment, proliferation, and differentiation assay using osteoblast cells.

## 2. Experimental Methods

### 2.1. Surface Topography Control of Ti Alloy

Titanium-40Niobium-6Zirconium alloy (TNZ) was manufactured by classic forging process and cut into the rectangular shape (10 mm × 10 mm ×2 mm) for the evaluations. Pure Ti and Nb with the purity of 99% (Alfa Aesar, USA) were used to study the mechanism of nanostructure formation on a metal surface via TIPS treatment as control groups. All the samples were ultrasonically cleaned in ethanol for 10 min before the fabrication and evaluation. To confirm a nano-topographical change of TNZ surface depending on the target metal ion sources in TIPS system, two different metal targets, niobium (Nb) and tantalum (Ta), were used. Nb and Ta targets (75-mm diameter, 5-mm thickness, 99.99% purity, Avention, Korea) were placed in a direct current (DC) magnetron sputtering gun (Ultech Co., Ltd., Daegu, Korea). Prior to the sputtering process, the vacuum chamber was sequentially pumped down to 5 × 10^−4^ Pa with rotary pumps followed by diffusion pumps. To generate numerous Nb or Ta ions for each Nb ion or Ta ion etching process, 65 W of target power were directly given to the targets. The working temperature and pressure of the process were maintained at 0.6 Pa and 25 °C, respectively. Two kinds of TIPS processes using Nb target (Nb-TIPS) and Ta target (Ta-TIPS) were applied on Pure Ti, TNZ, and Pure Nb surfaces with a negative substrate bias voltage ranging from 500 to 2000 V for 10 min to fabricate various surface morphologies.

### 2.2. Surface Characterization

The surface morphology observations were performed using field-emission scanning electron microscopy (FE-SEM; JSM-6330F, JEOL, Japan), and the surface topography such as depth and width of formed nanopatterns was analyzed by ImageJ software. A gallium (Ga) focused-ion beam was used to prepare STEM samples under a Ga^+^ ion current of 20 nA. The compositional analysis of nano-structured TNZ surfaces were carried out by high-resolution scanning transmission electron microscopy (STEM; JEM-2100F, JEOL, Japan) at 200 kV. Both Nb and Ta TIPS-treated TNZ with the condition of 2 kV substrate negative bias voltage were chosen as representative samples for compositional analysis. The Nb and Ta distribution profile on the TNZ surface was investigated by the energy-dispersive X-ray spectroscopy (EDS) with elemental spot analysis mode in STEM. The cross-sectional images of all the experimental groups were obtained by FE-SEM equipped with focused ion beam (FIB) milling (FIB/FE-SEM; AURIGA, Carl Zeiss, Germany). The hydrophilicity of Flat, Nb-, and Ta-TIPS–treated TNZ were measured three times per each group (n = 3) using a sessile drop method with distilled water and quantified using ImageJ software.

### 2.3. In Vitro Cellular Assays

The biological properties were evaluated using osteoblast cells (MC3T3, ATCC, CRL-2593, USA). The osteoblast cells were cultured in α-minimum essential medium (α-MEM; Welgene, Gyeongbuk, South Korea) supplemented with 10% fetal bovine serum and 1% penicillin-streptomycin, and incubated in a humidified incubator with 5% CO_2_ at 37 °C. The in vitro cellular assays including cell attachment, proliferation, and differentiation were conducted on the surfaces of TNZ surfaces etched by Nb (Nb-TIPS) and Ta ion (Ta-TIPS) under conditions of 5% CO_2_ at 37 °C. Osteoblastic cells were seeded on each surface of the experimental and control groups (Flat TNZ) at a density of 3, 2, and 1 × 10^4^ cells/mL for cell attachment, proliferation, and differentiation tests, respectively.

The cellular attachment on the surfaces of samples were characterized by CLSM. After 3 h of culturing, the adhered cells were fixed by 4% paraformaldehyde (Sigma-Aldrich, St. Louis, Mo, USA) and washed in PBS. The rinsed samples were permeabilized with 0.1% Triton (Sigma-Aldrich, St. Louis, Mo, USA) in PBS for 5 min. Nonspecific binding sites were blocked using 1% bovine serum albumin (BSA; Sigma-Aldrich, St. Louis, Mo, USA) in PBS for 30 min. Cell nuclei and F-actin were stained with 4′,6diamidino-2-phenylindole (DAPI; ProLong Gold antifate reagent with DAPI, Invitrogen, Carlsbad, CA, USA) and fluorescent phalloidin (Alexa Fluor 555 phalloidin, Invitrogen, USA), respectively. The surface coverage rate of osteoblast cells on each surface of samples were measured using ImageJ software (NIH, Bethesda, MD, Maryland, USA).

The cell proliferation test was performed by a methoxyphenyl tetrazolium salt (MTS, Promega, Madison, WI, USA) assay with 3-(4,5-dimethylthiazol-2-yl)-5-(3-carboxymethoxyphenyl)-2-(4-sulfophenyl)-2H-tetrazolium as a substrate for mitochondrial reduction. After 1 and 3 days of culturing, the volume of formazan product was measured at 490 nm to confirm viability of cells on the surfaces.

The cell differentiation of osteoblasts at an early stage was investigated by alkaline phosphatase (ALP) activity assay after 14 days of culturing. To accelerate the cell differentiation, β-GP of 10 mM and ascorbic acid of 50 mg/mL were added to the culture medium to induce accelerated cell differentiation after 24 h of culturing, and the culture medium was refreshed every three days. The ALP activity was quantified by a micro reader at 405 nm (Model 550, Bio-Rad Laboratories, Contra Costa County, CA, USA).

### 2.4. Statistical Analysis

All experiments were conducted on three samples per group. Statistical analysis was performed by the Statistical Package for the Social Sciences (SPSS 23, SPSS Inc., Chicago, IL, USA) software, and all experimental data were converted to mean ± standard deviation. The normality of the variables was assessed using the Shapiro−Wilk test, and the statistical analysis was performed by one-way analysis of variance followed by Tukey’s post-hoc comparison. A *p*-value of <0.05 was deemed significant.

## 3. Results

### 3.1. Surface Characterization

**Figure 1:**
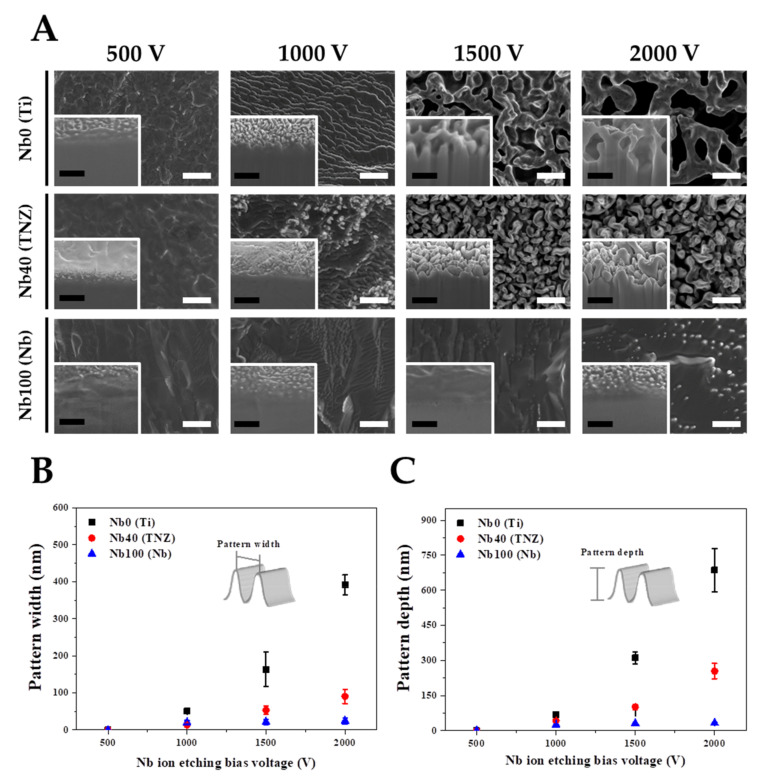
Surface characterization of pure Ti, TNZ alloys, and pure Nb etched by Nb ion (Nb-TIPS) as a function of substrate bias voltage; (**A**) surface and cross-sectional images, (**B**) width, and (**C**) depth of nanopatterns (both white and black scale bars in FE-SEM and FIB images indicate 500 nm).

**Figure 2:**
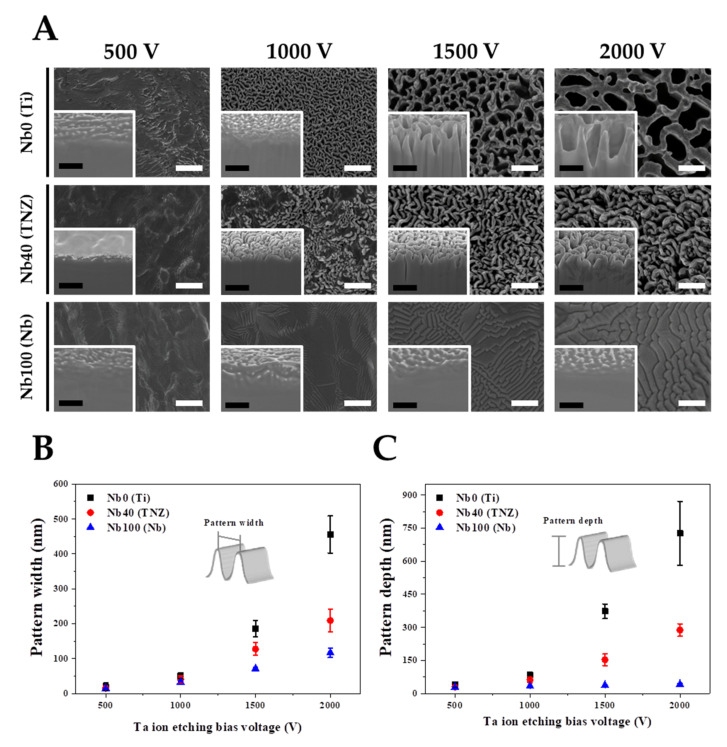
Surface characterization of pure Ti, TNZ alloys, and pure Nb etched by Ta ion (Ta-TIPS) as a function of substrate bias voltage; (**A**) surface and cross-sectional images, (**B**) width, and (**C**) depth of nanopatterns (both white and black scale bars in FE-SEM and FIB images indicate 500 nm).

Various experimental parameters were considered in order to understand the nano-topographical changes on the TNZ surfaces after Nb- or Ta-TIPS. The experimental variables applied in the TIPS system are as follows: (1) an applied negative substrate bias voltage ($V_{sub}$) ranging from 500 to 2000 V, (2) substrate materials: pure Ti (Nb content 0%), TNZ (Nb content 40%), and pure Nb (Nb content 100%), and (3) physical properties of target materials (Nb and Ta) as shown in Table 1. Changes in nano-topography induced by modulating the experimental variables were observed using FE-SEM and FIB (Figure 1). The lack of nanostructures in Nb- and Ta-TIPS for $V_{sub}\leq$ 500 V resulted from insufficient voltage for etching of the substrate via cation acceleration. The nanostructures comprising the Nb-TIPS–treated pure Ti changed significantly when $V_{sub}$ increased gradually from 500 to 2000 V (Figure 1B,C). The average widths and depths of the nanopatterns increased proportionally to the increase in $V_{sub}$ (Widths of 0, 51, 163, and 391 nm and corresponding depths of 0, 68, 311, and 686 nm, respectively, were obtained). However, the width and depth of the nanopatterns comprising the TNZ alloy with ~40% of Nb element in the matrix were lower than those of pure Ti over the entire $V_{sub}$ region (the average widths and depths of the nanopatterns are 0, 14, 54, and 90 nm and 0, 43, 101, and 255 nm, respectively). No topographical changes were observed for pure Nb (Nb content 100%), irrespective of the $V_{sub}$ value. For the Nb-TIPS, in all the experimental groups except for the pure Nb material, the size and depth of the nanostructures increased with increasing $V_{sub}$. Surface and cross-sectional images of Ta-TIPS–treated pure Ti, TNZ, and pure Nb are shown in Figure 2. In the case of pure Ti (Nb content 0%), nanostructures started to form at $V_{sub}$ ≥ 1000 V. The widths and depths of the generated nanostructures (0, 51, 186, and 456 nm and 0, 85, 373, 727 nm, respectively) increased with increasing $V_{sub}$ ranging from 500 to 2000 V. For TNZ alloys with 40% of Nb element in the matrix, the average nanopattern widths of 0, 40, 127, and 210 nm and depths of 0, 62, 153, and 288 nm were obtained after Ta-TIPS. The average nanopattern widths of pure Nb (Nb content 100%) (0, 32, 71, and 116 nm), which were the lowest among the widths of the three substrate materials, increased with increasing $V_{sub}$. Nano-wrinkle structures rather than nanopattern structures occurred in pure Nb, and the depth of these wrinkles changed only slightly with increasing $V_{sub}$. For both Nb- and Ta-TIPS–treated materials, the size of the nanostructures comprising the substrate materials was inversely proportional to the Nb content of the matrix. For TNZ alloys, the threshold voltage required to create nanostructures ($V_{th}$) decreased from 1500 (Nb-TIPS) to 1000 V (Ta-TIPS). This indicated that, compared with Nb, Ta ions were more effective in etching the substrate materials due to the high atomic weight of Ta (see Table 1 and Figure S1).

### 3.2. Surface Chemistry and Hydrophilicity

**Figure 3:**
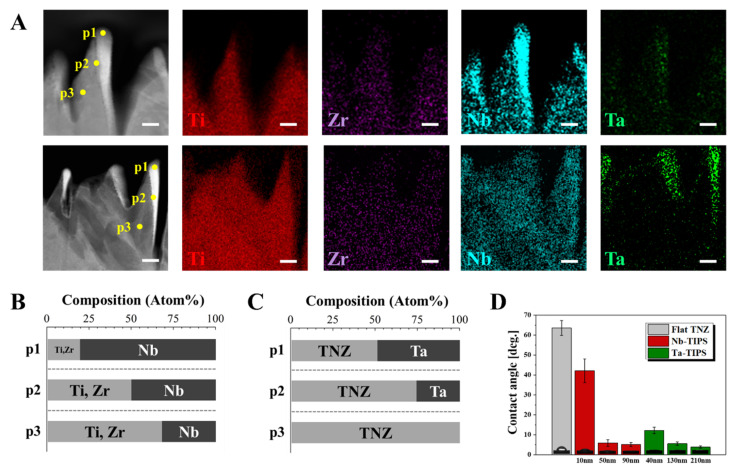
Cross-sectional scanning transmission electron microscopy image of TNZ surfaces etched by (**A**) Nb- (upper) and Ta-TIPS (lower) with energy-dispersive X-ray spectroscopy mapping (Ti, Zr, Nb, and Ta elements). Chemical compositions at three regions of TNZ nanostructures etched by (**B**) Nb- and (**C**) Ta-TIPS (p1: top, p2: middle, and p3: bottom). (Scale bar = 50 nm). (**D**) Water contact angles of Flat, Nb-, and Ta-TIPS–treated TNZ. Inset images denotes optical images of water droplets on each surface.

STEM and EDS images revealed physicochemical changes in the nanostructured TNZ surface (Figure 3 and Figure S2). The flat TNZ surface became a deep and sharp nano-peak structure comprising Nb or Ta atoms, which are the etching agents present at the top region of the nano-peaks after Nb- or Ta-TIPS. For Nb-TIPS–treated TNZ, Nb atoms were present at the top of the peaks. A larger number (2.7 times more) of these atoms occurred at the top part (p1) than in the TNZ matrix (p3) as shown in Figure 3B. The number of incorporated Nb atoms decreased from the surface toward the substrate. This confirmed that Ta atoms (bright color part) were stably incorporated into the matrix, as illustrated in TEM images of the Ta-TIPS–treated TNZ surface. EDS point analysis indicated that the Ta atoms occupied ~170 nm of the total nanostructure. In addition, the number of these atoms decreased toward the substrate (p1-> p2-> p3, 50%-> 25%-> 0%), as described in Figure 3C.

The hydrophilicity, which plays an important role in bioactivity, was measured using a sessile drop method. Each experimental group was named based on the mean value of each nanopattern width as a function of the $V_{sub}$ (except for 500 V, which yielded no nanostructure on the TNZ surfaces). Nb-TIPS–treated TNZ exhibited nanopatterns with widths of 10 nm, 50 nm, and 90 nm, and Ta-TIPS–treated TNZ exhibited nanopatterns with larger widths of 40 nm, 130 nm, and 210 nm. The average contact angle of the Flat TNZ (control group) was 63°. The contact angles of Nb-TIPS–treated TNZ groups (10, 50, and 90 nm) were 42°, 6°, and 5°, respectively. The Ta-TIPS–treated TNZ groups (40, 130, and 210 nm) were characterized by average contact angle values of 12°, 6°, and 4°, respectively. As a result, the hydrophilicity of particular groups in the nanostructured TNZ (nanopattern width ≥ 40 nm) was considerably (over five times) greater than that of the Flat TNZ.

### 3.3. In Vitro Osteoblastic Cell Responses

**Figure 4:**
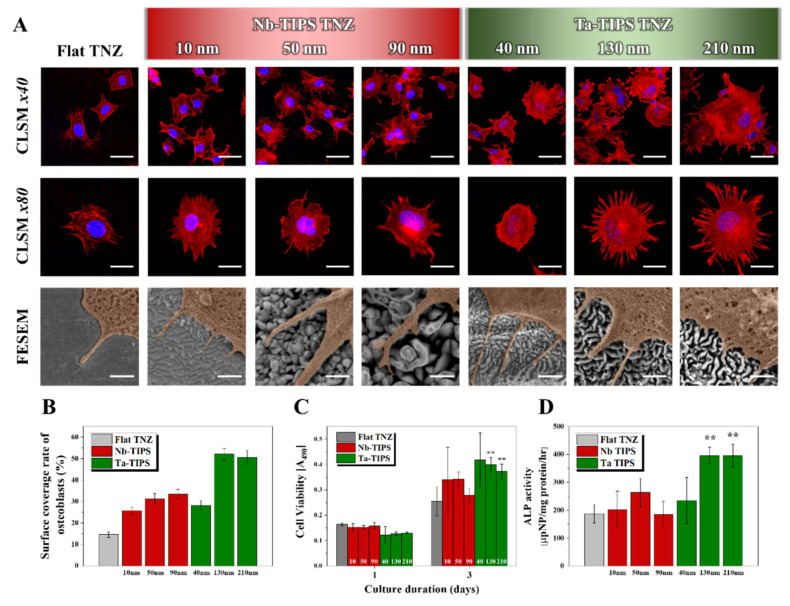
In vitro osteoblast cell tests of Flat, Nb- (10, 50, and 90 nm), and Ta-TIPS (40, 130, and 210 nm) treated TNZ. (**A**) Low (upper) and high (lower) magnification CLSM images of adhered cells on the surfaces. (**B**) The surface coverage rate of osteoblast cells quantified from CLSM images. (**C**) Cell proliferation, and (**D**) differentiation (ALP activity) of osteoblast cells after 1, 3, and 6 days and 14 days, respectively (** *p* < 0.005). The white scale bars in the top, middle, and bottom lines indicate 50 μm, 25 μm, and 500 nm, respectively.

Osteoblastic cell adhesion, proliferation, and differentiation were measured to confirm the cytocompatibility of the nanostructured TNZ surfaces (Figure 4). In the osteoblastic cell attachment test (see Figure 4A), the actin filament (red) and nuclei (blue) of the cells were stained and observed by CLSM and FE-SEM. The shapes of osteoblasts adhered on the TNZ surfaces differed only slightly from that of the control group even after the Nb-TIPS treatment (Figure 4A CLSM × 80 images). However, significant shape changes occurred in the Ta-TIPS–treated TNZ groups. In particular, compared with Flat TNZ, the 130 and 210 nm groups was characterized by a larger area of attached osteoblasts, and an activated cytoskeleton. The surface coverage rate of the osteoblasts was determined from CLSM images and then used to quantify the area of osteoblast cells attached to each surface. The experimental groups with nanopattern widths ≥130 nm (Ta-TIPS TNZ 130 and 210 nm groups) exhibited excellent osteoblastic cell adhesion levels, which were 3.5 and 3.4 times higher than that of Flat TNZ (Figure 4B). The viability of the cells was evaluated through a cell proliferation test. After 1 day of culturing, all the groups exhibited similar levels of cell viability regardless of the nanostructure size. After 3 days of cell culturing, no improvement in the cytocompatibility of the Nb-TIPS–treated TNZ (relative to that of Flat TNZ) was observed. In contrast, the 130 and 210 nm groups exhibited enhanced proliferation abilities that were 157% and 146% higher, respectively, than that of the control group (Figure 4C). Osteoblast cell differentiation was evaluated on cells cultured for 14 days under the physiological condition. No enhancement in cytocompatibility compared with that of Flat TNZ was observed for the groups with nanopattern widths of <100 nm (Nb-TIPS 10, 50, and 90 nm and Ta-TIPS 40 nm). However, the cytocompatibility of the 130 and 210 nm groups was over two times higher than that of the control group (Figure 4D).

## 4. Discussion

Ti-based alloys are promising metallic biomaterials with excellent mechanical strength for effectively supporting or replacing damaged tissue. However, the use of these materials is restricted due to their poor bio-affinity in the early stages of implantation, which leads to failure of medical implants and, consequently, re-operation. In this study, we applied TIPS technology to TNZ materials with the aim of imparting excellent cytocompatibility to the implant surface. Various experimental parameters were employed in order to generate nanostructures of different sizes (10–210 nm). These parameters included the substrate materials (pure Ti, TNZ alloy, and pure Nb), $V_{sub}$ (500–2000 V), the negative bias voltage applied to the substrate, and target materials (Nb or Ta).

Firstly, the $V_{sub}$ played an important role in determining the etching efficiency of the substrate materials. The Nb or Ta cations emitted from the target during each Nb- or Ta-TIPS process were accelerated by an extremely high negative bias voltage applied to the substrate, leading to the formation of a collision cascade on the outermost surface of the substrate. The size of the nanopatterns increased with increasing $V_{sub}$ regardless of the substrate type. Secondly, the physical properties such as atomic mass and density of the substrate materials are closely correlated with the degree of nanostructure formation. Theoretically, the atomic mass of substrate materials is inversely related to the sputtering efficiency (see Equation (1)). The ion implantation efficiency is inversely proportional to the density of the substrate materials, indicating that the resistance to metal ion etching increases with increasing density of the substrate (see Equation (2)). The density of Nb is approximately two times higher than that of Ti, suggesting that an increase in the Nb content of the metal matrix increased the resistance to the Nb or Ta ion etching. Furthermore, the value of $m_{s}$ increased with increasing Nb content (see Equation (1)), leading to a decrease in the sputtering yield (TIPS yield).

(1) $$\mathrm{Sputtering}\;\mathrm{yield}=3.56\;\alpha \;\left[\frac{Z_{i}Z_{s}}{Z_{i}{}^{\frac{2}{3}}+Z_{s}{}^{\frac{2}{3}}}\right]\left[\frac{m_{i}}{m_{i}+m_{s}}\right]\frac{S_{n}\left(E\right)}{U_{o}}$$

(2) $$R_{P}=\frac{3m_{i}}{3m_{i}+m_{s}}R$$

where, $Z_{i}$ and $Z_{s}$ are the respective atomic numbers of injected ions and substrate materials. $m_{i}$ and $m_{s}$ represent the atomic mass of ions and substrate materials, respectively. $U_{o}$, $S_{n}$(E), R, and $R_{p}$ represent the surface binding energy of the substrate material, reduced stopping power, the ion range of the implantation, and the projection range, respectively [21,22]. Thirdly, the atomic mass of the sputtering target materials affects the sputtering-based ion etching efficiency. The atomic mass of the metal cation emitted from sputter targets and the sputtering yield are proportional as illustrated in Equation (1). The effectiveness of the metal-cation etching performed on TNZ during the sputtering process increases with increasing atomic mass of the target materials, as described in Figure S1. The depth at which ions can be injected into the substrate matrix (R and $R_{P}$) is proportional to the atomic mass of the target ion as presented in Equation (2). For example, consider the pure Ti, TNZ, and pure Nb groups under a given set of experimental conditions. Compared with the Nb-TIPS treatment, the Ta-TIPS treatment yielded larger nanostructure sizes of each group. This is especially true for the TNZ surfaces where Nb- and Ta-TIPS generated nanopatterns with widths of 90 and 210 nm, respectively (Figure 1B and Figure 2B). The size of the nanopatterns, which increased approximately two-fold, resulted from the approximately two-fold increase in the atomic mass of the target material (from Nb 92.91 to Ta 180.95; see Table 1). The topography of the pure Nb remained unchanged after Nb-TIPS, even when the maximum voltage of 2000 V was applied. The Ta-TIPS treatment generated nano-wrinkle structures on the pure Nb surface, owing to the higher atomic mass of Ta compared with that of Nb. That is, the atomic mass of the target materials directly affects the etching efficiency of the substrate materials.

Nb or Ta atoms were uniformly distributed at the nano-level in the TNZ matrix as observed via TEM and EDS analysis. Nano-roughness is caused by the difference in local surface erosion rates during the ion etching process [23]. The Nb or Ta ions that collide with the outermost surface of TNZ are strongly incorporated, thereby forming a mechanically robust pair with TNZ without a physical interface. The hydrophilicity of specific experimental groups increased significantly after the Nb- or Ta-TIPS (nanopattern width ≥ 40 nm) process. The dramatic changes in the surface wettability of the nanostructured TNZ surfaces are mainly attributed to the surface nano-roughness. The gap between surface patterns can minimize the formation of air pockets and facilitate the entering and filling of the pattern with water via the capillary effect, which increases the surface hydrophilicity substantially. On the contrary, according to the hydrophilicity of Flat TNZ, it exhibits a near hydrophobic surface property, showing water contact angle larger than 60°. This property is an important factor that indirectly represents the bio-affinity with substances such as proteins, blood, and cells when biomaterials are implanted in the body [24,25].

The biocompatibility was confirmed through evaluations of the osteoblast cell attachment, proliferation, and differentiation. The cell adhesion property was enhanced in all the nanostructured TNZ groups, owing to the formation of focal adhesions in which cells exhibit strong interactions with nanostructured surfaces [26,27,28]. Such surfaces can trigger biologically active cellular responses by controlling integrin-dependent cell adhesion signaling pathways. The results suggested that a specific morphological condition (nanopattern width ≥ 130 nm) was required for achieving excellent osteoblast cell adhesion as well as cell proliferation and differentiation [29]. 

## 5. Conclusions

In this work, we successfully fabricated various nano-topography on the surfaces of TNZ by varying the experimental conditions of TIPS. The surface of TNZ alloys was physicochemically modified with the formation of a mechanically robust nanostructured pair between target metal atom and TNZ matrix. Nanostructured TNZ surfaces exhibited superhydrophilicity which leads to the enhancement of osteoblastic cell adhesion, proliferation, and differentiation ability of biomaterials. These achievements imply that TIPS treatment has high potential to enhance clinical performance of metallic bio-implants. 

## Figures and Tables

**Table 1:** Intrinsic physical property of Ti, Nb, and Ta.

Materials	Atomic Number	Atomic Weight	Density (g/cm^3^)
Titanium (Ti)	22	47.87	4.51
Niobium (Nb)	41	92.91	8.57
Tantalum (Ta)	73	180.95	16.65

## Data Availability

Not included.

## Supplementary Materials

The following are available online at https://www.mdpi.com/article/10.3390/nano11061507/s1, Figure S1: The width of nano-patterns formed on TNZ substrate as a function of atomic mass ratio between target and substrate materials. Figure S2: EDS point analysis spectra of (A) Nb- and (B) Ta-TIPS TNZ.

## References

[1] Kaur M., Singh K. (2019). Review on titanium and titanium based alloys as biomaterials for orthopaedic applications. Mater. Sci. Eng. C.

[2] Chouirfa H., Bouloussa H., Migonney V., Falentin-Daudré C. (2019). Review of titanium surface modification techniques and coatings for antibacterial applications. Acta Biomater..

[3] Jung H.-D., Jang T.-S., Wang L., Kim H.-E., Koh Y.-H., Song J. (2015). Novel strategy for mechanically tunable and bioactive metal implants. Biomaterials.

[4] Lee H., Lee M.-K., Cheon K.-H., Kang I.-G., Park C., Jang T.-S., Han G., Kim H.-E., Song J., Jung H.-D. (2021). Functionally assembled metal platform as lego-like module system for enhanced mechanical tunability and biomolecules delivery. Mater. Des..

[5] Guillory R.J., Bowen P.K., Hopkins S.P., Shearier E.R., Earley E.J., Gillette A.A., Aghion E., Bocks M.L., Drelich J.W., Goldman J. (2016). Corrosion Characteristics Dictate the Long-Term Inflammatory Profile of Degradable Zinc Arterial Implants. ACS Biomater. Sci. Eng..

[6] He Y., Zhang Y., Meng Z., Jiang Y., Zhou R. (2017). Microstructure evolution, mechanical properties and enhanced bioactivity of Ti-Nb-Zr based biocomposite by bioactive calcium pyrophosphate. J. Alloys Compd..

[7] Kim K.M., Kim H.Y., Miyazaki S. (2020). Effect of Zr Content on Phase Stability, Deformation Behavior, and Young’s Modulus in Ti–Nb–Zr Alloys. Materials.

[8] Rao S., Astaneh S.H., Villanueva J., Silva F., Takoudis C., Bijukumar D., Souza J.C., Mathew M.T. (2019). Physicochemical and in-vitro biological analysis of bio-functionalized titanium samples in a protein-rich medium. J. Mech. Behav. Biomed. Mater..

[9] Xiao M., Chen Y., Biao M., Zhang X., Yang B. (2017). Bio-functionalization of biomedical metals. Mater. Sci. Eng. C.

[10] Zhou J., Yang Y., Frank M.A., Detsch R., Boccaccini A.R., Virtanen S. (2016). Accelerated Degradation Behavior and Cytocompatibility of Pure Iron Treated with Sandblasting. ACS Appl. Mater. Interfaces.

[11] Modic M., Kovač J., Nicholls J.R., Kos Š., Serša G., Cvelbar U., Walsh J.L. (2019). Targeted plasma functionalization of titanium inhibits polymicrobial biofilm recolonization and stimulates cell function. Appl. Surf. Sci..

[12] Damiati L., Eales M.G., Nobbs A., Su B., Tsimbouri P.M., Salmeron-Sanchez M., Dalby M. (2018). Impact of surface topography and coating on osteogenesis and bacterial attachment on titanium implants. J. Tissue Eng..

[13] Bello D.G., Fouillen A., Badia A., Nanci A. (2017). A nanoporous titanium surface promotes the maturation of focal adhesions and formation of filopodia with distinctive nanoscale protrusions by osteogenic cells. Acta Biomater..

[14] Okulov I.V., Joo S.-H., Okulov A., Volegov A.S., Luthringer B., Willumeit-Römer R., Zhang L., Mädler L., Eckert J., Kato H. (2020). Surface Functionalization of Biomedical Ti-6Al-7Nb Alloy by Liquid Metal Dealloying. Nanomaterials.

[15] Jang T.-S., Kim S., Jung H.-D., Chung J.-W., Kim H.-E., Koh Y.-H., Song J. (2016). Large-scale nanopatterning of metal surfaces by target-ion induced plasma sputtering (TIPS). RSC Adv..

[16] Kim J., Lee H., Jang T.-S., Kim D., Yoon C.-B., Han G., Kim H.-E., Jung H.-D. (2021). Characterization of Titanium Surface Modification Strategies for Osseointegration Enhancement. Metals.

[17] Park C., Seong Y.-J., Kang I.-G., Song E.-H., Lee H., Kim J., Jung H.-D., Kim H.-E., Jang T.-S. (2019). Enhanced Osseointegration Ability of Poly(lactic acid) via Tantalum Sputtering-Based Plasma Immersion Ion Implantation. ACS Appl. Mater. Interfaces.

[18] Park C., Park S., Kim J., Han A., Ahn S., Min S.-K., Jae H.J., Chung J.W., Lee J.-H., Jung H.-D. (2020). Enhanced endothelial cell activity induced by incorporation of nano-thick tantalum layer in artificial vascular grafts. Appl. Surf. Sci..

[19] Vandana U., Nancy D., Sabareeswaran A., Remya N., Rajendran N., Mohanan P. (2021). Biocompatibility of strontium incorporated ceramic coated titanium oxide implant indented for orthopaedic applications. Mater. Sci. Eng. B.

[20] Lu T., Wen J., Qian S., Cao H., Ning C., Pan X., Jiang X., Liu X., Chu P.K. (2015). Enhanced osteointegration on tantalum-implanted polyetheretherketone surface with bone-like elastic modulus. Biomaterials.

[21] Li X., An Y., Wei Y., Du H., Hou L., Guo C., Qu H., Wang Y. (2015). Influence of Surface Nanocrystallization on Ti Ion Implantation of Pure Iron. J. Mater. Sci. Technol..

[22] Jain I., Agarwal G. (2011). Ion beam induced surface and interface engineering. Surf. Sci. Rep..

[23] Muñoz-García J., Vázquez L., Castro M., Gago R., Redondo-Cubero A., Moreno-Barrado A., Cuerno R. (2014). Self-organized nanopatterning of silicon surfaces by ion beam sputtering. Mater. Sci. Eng. R Rep..

[24] Cheon K.-H., Park C., Kang M.-H., Kang I.-G., Lee M.-K., Lee H., Kim H.-E., Jung H.-D., Jang T.-S. (2021). Construction of tantalum/poly(ether imide) coatings on magnesium implants with both corrosion protection and osseointegration properties. Bioact. Mater..

[25] Park C., Lee S.-W., Kim J., Song E.-H., Jung H.-D., Park J.-U., Kim H.-E., Kim S., Jang T.-S. (2019). Reduced fibrous capsule formation at nano-engineered silicone surfaces via tantalum ion implantation. Biomater. Sci..

[26] Kim J., Bae W.-G., Choung H.-W., Lim K.T., Seonwoo H., Jeong H.E., Suh K.-Y., Jeon N.L., Choung P.-H., Chung J.H. (2014). Multiscale patterned transplantable stem cell patches for bone tissue regeneration. Biomaterials.

[27] Yao M., Cheng S., Zhong G., Zhou J., Shao H., Ma L., Du C., Peng F., Zhang Y. (2021). Enhanced osteogenesis of titanium with nano-Mg(OH)2 film and a mechanism study via whole genome expression analysis. Bioact. Mater..

[28] Necula M.G., Mazare A., Ion R.N., Ozkan S., Park J., Schmuki P., Cimpean A. (2019). Lateral Spacing of TiO2 Nanotubes Modulates Osteoblast Behavior. Materials.

[29] Kim J., Kim H.N., Lim K.-T., Kim Y., Pandey S., Garg P., Choung Y.-H., Choung P.-H., Suh K.-Y., Chung J.H. (2013). Synergistic effects of nanotopography and co-culture with endothelial cells on osteogenesis of mesenchymal stem cells. Biomaterials.

