# Supplementary material for: Nano-Topographical Control of Ti-Nb-Zr Alloy Surfaces for Enhanced Osteoblastic Response
Source: Nanomaterials (Basel). 2021 Jun 7;11(6):1507. doi: 10.3390/nano11061507 (PMC8229642; doi:10.3390/nano11061507)
Supplement: Supplementary file 1 [file nanomaterials-11-01507-s001.zip › nanomaterials-1215464-supplementary.pdf]

nanomaterials

Communication

Nano-Topographical Control of Ti-Nb-Zr Alloy Surfaces for Enhanced Osteoblastic Response

Min-Kyu Lee [1,2], Hyun Lee [3,4], Hyoun-Ee Kim [1], Eun-Jung Lee [5], Tae-Sik Jang [6,*] and Hyun-Do Jung [3,4,*]

1 Department of Materials Science and Engineering, Seoul National University, Seoul 08826, Korea; elzkdnem@snu.ac.kr (M.-K.L.); kimhe@snu.ac.kr (H.-E.K.)
2 Department of Materials Science and Engineering and Querrey-Simpson Institute for Bioelectronics, Northwestern University, Evanston, IL 60208, USA
3 Department of Biomedical-Chemical Engineering, Catholic University of Korea, Bucheon14662, Korea; leeh0520@catholic.ac.kr
4 Department of Biotechnology, The Catholic University of Korea, Bucheon 14662, Korea
5 Department of Nano-Biomedical Science & BK21 PLUS NBM Global Research Center for Regenerative Medicine, Dankook University, Cheonan 31116, Korea; ejlee79@dankook.ac.kr
6 Department of Materials Science and Engineering, Chosun University, Gwangju 61452, Korea
* Correspondence: tsjang@chosun.ac.kr (T.-S.J.); hdjung@catholic.ac.kr (H.-D.J.)

Citation: Lee, M.-K.; Lee, H.; Kim, H.-E.; Lee, E.-J.; Jang, T.-S.; Jung, H.-D. Nano-Topographical Control of Ti-Nb-Zr Alloy Surfaces for Enhanced Osteoblastic Response. *Nanomaterials* **2021**, *11*, 1507. https://doi.org/10.3390/nano11061507

Academic Editors: Hicham Fenniri and Junghwan Lee

Received: 24 April 2021
Accepted: 2 June 2021
Published: 7 June 2021

Publisher's Note: MDPI stays neutral with regard to jurisdictional claims in published maps and institutional affiliations.

Copyright: © 2021 by the authors. Licensee MDPI, Basel, Switzerland. This article is an open access article distributed under the terms and conditions of the Creative Commons Attribution (CC BY) license (http://creativecommons.org/licenses/by/4.0/).

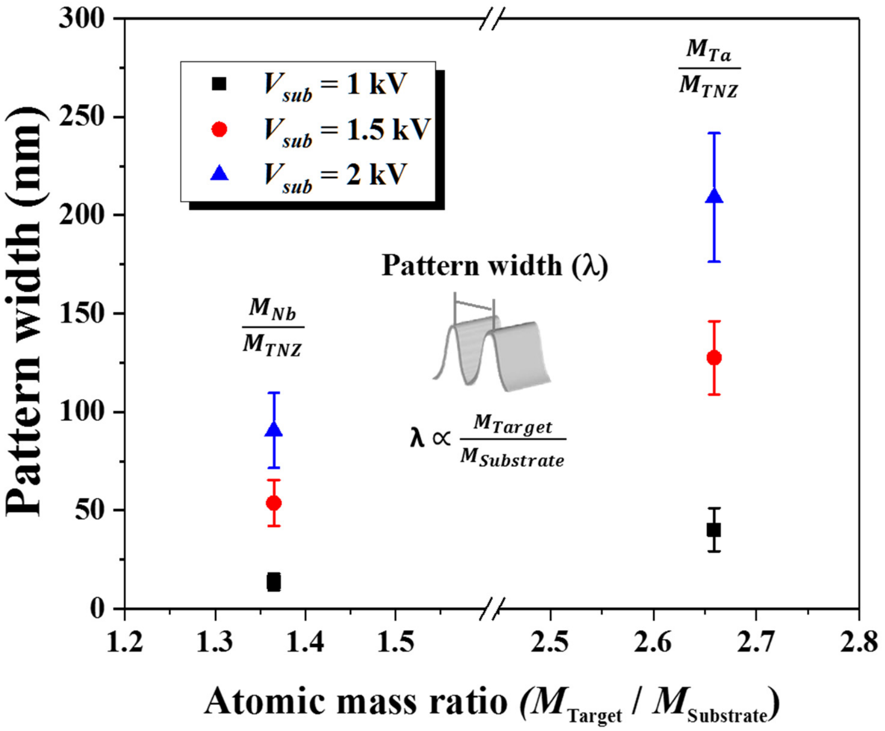

Figure S1. The width of nano-patterns formed on TNZ substrate as a function of atomic mass ratio between target and substrate materials.

A

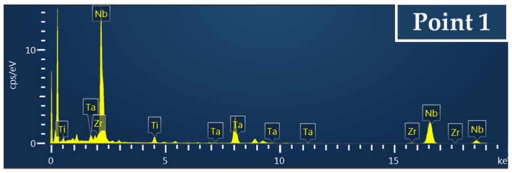

Element	Line Type	k Factor type	k Factor ty pe	Absorption Correction	Wt%	Wt% Sigm a	Atomic %
Ti	K series	1.090		1.00	5.97	0.16	11.28
Zr	K series	3.211		1.00	1.91	0.22	1.90
Nb	K series	3.717		1.00	86.17	0.48	83.86
Ta	L series	2.135		1.00	5.95	0.43	2.97
Total:					100.00		100.00

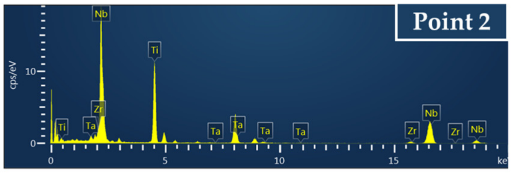

Element	Line Type	k Factor	k Factor ty pe	Absorption Correction	Wt%	Wt% Sigm a	Atomic %
Ti	K series	1.090		1.00	31.90	0.35	48.11
Zr	K series	3.211		1.00	3.54	0.22	2.80
Nb	K series	3.717		1.00	61.64	0.44	47.92
Ta	L series	2.135		1.00	2.92	0.35	1.17
Total:					100.00		100.00

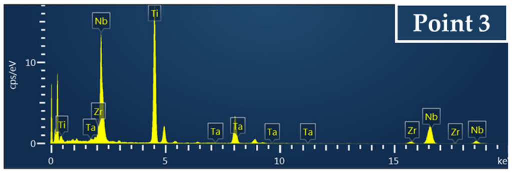

Elemen t	Line Type	k Factor	k Factor t ype	Absorption Correction	Wt%	Wt% Sig ma	Atomic %
Ti	K series	1.090		1.00	49.71	0.43	65.94
Zr	K series	3.211		1.00	4.02	0.23	2.80
Nb	K series	3.717		1.00	45.12	0.44	30.86
Ta	L series	2.135		1.00	1.15	0.31	0.40
Total:					100.00		100.00

B

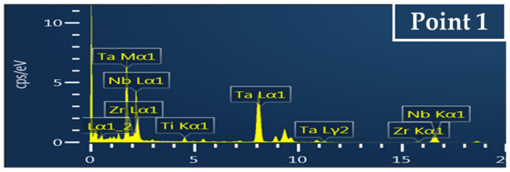

Elemen t	Line Type	k Factor	k Factor t ype	Absorption Correction	Wt%	Wt% Sig ma	Atomic %
Ti	K series	1.090		1.00	2.22	0.07	6.14
Zr	K series	3.211		1.00	1.09	0.12	1.58
Nb	K series	3.717		1.00	31.07	0.34	44.27
Ta	L series	2.135		1.00	65.61	0.35	48.00
Total:					100.00		100.00

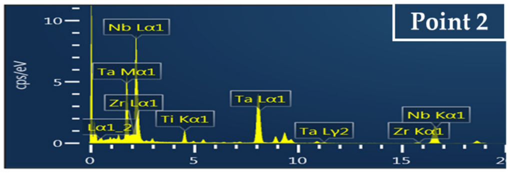

Elemen t	Line Type	k Factor	k Factor t ype	Absorption Correction	Wt%	Wt% Sig ma	Atomic %
Ti	K series	1.090		1.00	4.90	0.09	11.33
Zr	K series	3.211		1.00	1.67	0.13	2.02
Nb	K series	3.717		1.00	50.87	0.35	60.61
Ta	L series	2.135		1.00	42.56	0.35	26.04
Total:					100.00		100.00

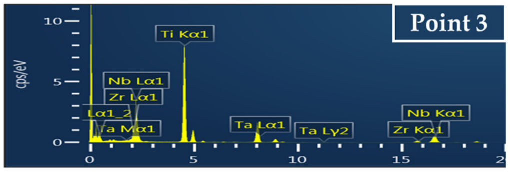

Elemen t	Line Type	k Factor	k Factor t ype	Absorption Correction	Wt%	Wt% Sig ma	Atomic %
Ti	K series	1.090		1.00	62.34	0.29	76.66
Zr	K series	3.211		1.00	5.34	0.15	3.45
Nb	K series	3.717		1.00	30.37	0.27	19.26
Ta	L series	2.135		1.00	1.95	0.19	0.63
Total:					100.00		100.00

Figure S2. EDS point analysis spectra of (**A**) Nb- and (**B**) Ta-TIPS TNZ.
